# Antioxidant Activity Evaluation of *Oviductus Ranae* Protein Hydrolyzed by Different Proteases

**DOI:** 10.3390/molecules26061625

**Published:** 2021-03-15

**Authors:** Shihan Wang, Yuanshuai Gan, Xinxin Mao, Hong Kan, Nan Li, Changli Zhang, Zhihan Wang, Yongsheng Wang

**Affiliations:** 1 College of Chinese Medicinal Materials, Jilin Agricultural University, Changchun 130118, China; maomaoheniunai@126.com (X.M.); kanhong@vip.163.com (H.K.); 2 School of Pharmaceutical Sciences, Jilin University, Changchun 130021, China; ganys18@mails.jlu.edu.cn (Y.G.); lin20@mails.jlu.edu.cn (N.L.); clzhang20@mails.jlu.edu.cn (C.Z.); 3 Department of Physical Sciences, Eastern New Mexico University, Portales, NM 88130, USA; zhihan.wang@enmu.edu

**Keywords:** *Oviductus Ranae*, protein, enzymolysis, antioxidants, degree of hydrolysis, nutraceuticals

## Abstract

As nutrition and a health tonic for both medicine and food, the protein content of *Oviductus Ranae* is more than 40%, making it an ideal source to produce antioxidant peptides. This work evaluated the effects of six different proteases (pepsin, trypsin, papain, flavourzyme, neutral protease and alcalase) on the antioxidant activity of *Oviductus Ranae* protein, and analyzed the relationship between the hydrolysis time, the degree of hydrolysis (DH) and the antioxidant activity of the enzymatic hydrolysates. The results showed that the antioxidant activity of *Oviductus Ranae* protein was significantly improved and the optimal hydrolysis time was maintained between 3–4 h under the action of different proteases. Among them, the protein hydrolysate which was hydrolyzed by pepsin for 180 min had the strongest comprehensive antioxidant activity and was most suitable for the production of antioxidant peptides. At this time, the DH, the DPPH radical scavenging activity, the absorbance value of reducing power determination and the hydroxyl radical scavenging activity corresponding to the enzymatic hydrolysate were 13.32 ± 0.24%, 70.63 ± 1.53%, 0.376 ± 0.009 and 31.96 ± 0.78%, respectively. Correlation analysis showed that there was a significant positive correlation between the hydrolysis time, the DH and the antioxidant activity of the enzymatic hydrolysates, further indicating that the hydrolysates of *Oviductus Ranae* protein had great antioxidant potential. The traditional anti-aging efficacy of *Oviductus Ranae* is closely related to the scavenging of reactive oxygen species, and its hydrolysates have better antioxidant capacity, which also provides support for further development of its traditional anti-aging efficacy.

## 1. Introduction

Reactive oxygen species (ROS) are byproducts produced by the normal metabolism of oxygen in organisms, mainly in the form of superoxide radical, hydroxyl radical, singlet oxygen and hydrogen peroxide [1,2]. The normal level of ROS plays an important role in cell signaling and homeostasis [3,4]. However, the excessive production of ROS will react with biological macromolecules such as DNA and protein, causing oxidative damage to cells and tissues, and further triggering a series of chronic diseases such as diabetes, arteriosclerosis, Parkinson’s disease, and Alzheimer’s disease [5,6]. Therefore, various forms of antioxidants have been widely studied to avoid the damage caused by excessive ROS. The chemically synthesized antioxidants, such as propyl gallate (PG) and tertiary butylhydroquinone (TBHQ), probably have potential toxicity and health-related risks, so natural antioxidants have attracted much attention [7,8,9]. Antioxidant peptides, as a kind of natural antioxidant, have been obtained from the enzymatic hydrolysates of milk protein, fish protein, bean protein and other food proteins [10,11,12,13,14,15]. Due to the high edible safety and a wide range of access methods, antioxidant peptides from food sources have gradually become a research hotspot in the fields of medicine and food [5,16].

*Oviductus Ranae* is the dried oviduct of female *Rana temporaria chensinensis* David, which is mainly produced in Changbai Mountain area, Jilin Province, China [17,18,19]. As a nutritional and health supplement for both medicine and food, *Oviductus Ranae* is rich in protein, sterol, unsaturated fatty acid and other nutrients [20,21,22,23,24,25]. Protein is the chief composition of *Oviductus Ranae*, which usually accounts for more than 40%, and contains eight essential amino acids [18,26]. As the representative component of *Oviductus Ranae*, the protein content is much higher than most typical high-protein foods (for example, in typical high-protein foods, the protein content of poultry meat, fish and eggs is approximately 25%, 20%, 15%, respectively), so it is considered an ideal source for the production of antioxidant peptides. Anti-aging is one of the important traditional efficacies of *Oviductus Ranae* [27,28], and the aging process is mostly closely related to the production of excess ROS. Antioxidants can inhibit the production of ROS and remove excessive ROS, keeping the content of ROS at normal levels. Therefore, the research related to the antioxidant activity of *Oviductus Ranae* protein is also a supplementary explanation for its traditional anti-aging efficacy.

Enzymatic hydrolysis is the most typical method to produce antioxidant peptides, which can release various characteristic structures (e.g., functional side groups, domains) with the antioxidant capacity in proteins to enhance antioxidant activity and the nutritional value of *Oviductus Ranae* protein. In the process of enzymatic hydrolysis, the type of protease and the degree of hydrolysis (DH) are closely related to antioxidant properties of the protein hydrolysate [29,30]. Due to different action sites of proteases, the type of protease significantly affects the cleavage pattern of peptide bonds in proteins [31]. For example, pepsin tends to hydrolyze peptide bonds whose N-terminus or C-terminus are aromatic amino acids (e.g., tryptophan and tyrosine) [32]; papain hydrolyzes the C-terminus of arginine and lysine in proteins, and can preferentially hydrolyze peptide bonds composed of amino acids with two carboxyl groups at the N-terminus or aromatic amino acids [33], and so on. In addition to the type of protease, the DH of the protein substrate during the enzymolysis process also affects the size of polypeptides, which in turn affects the amino acid composition of the polypeptide, resulting in changes in the antioxidant activity of the hydrolysate [34].

A proper protease to produce the antioxidant peptides is important for the further development and application of *Oviductus Ranae* protein antioxidant peptide-related products. The proteases selected in the study were six kinds of typical proteases in food production, including pepsin and trypsin extracted from animal stomach and pancreas, plant-derived papain, flavourzyme, neutral protease and alcalase produced by microbial fermentation. The six kinds of proteases were used to act on *Oviductus Ranae* protein and samples were taken at different hydrolysis times. By observing the changes of *Oviductus Ranae* protein in the process of hydrolysis and determining the DH and antioxidant activity of different samples, this work studied the effect of enzymatic hydrolysis of different proteases on the antioxidant activity of *Oviductus Ranae* protein, so as to preliminarily determine the antioxidant potential of the hydrolysate of *Oviductus Ranae* protein.

## 2. Results and Discussions

### 2.1. The Solubility of Oviductus Ranae Protein

**Figure 1:**
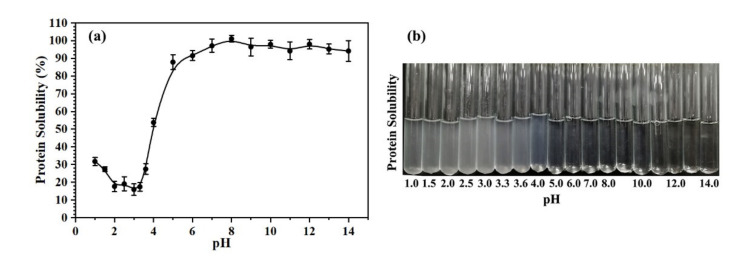
The solubility of *Oviductus Ranae* protein in different pH environments. (**a**) The solubility curve of *Oviductus Ranae* protein, (**b**) the solution phenomenon of *Oviductus Ranae* protein in different pH environments.

The *Oviductus Ranae* protein solution was adjusted to different pH values (pH range of 1.0–14.0), and the solution phenomenon after mixing is shown in Figure 1b. In the pH range of 1.0–4.0, the solution of *Oviductus Ranae* protein was obviously turbid due to a large amount of precipitation. The pH of the solution was closely related to the solubility of the protein, and the solubility curve of *Oviductus Ranae* protein changing with pH is shown in Figure 1a. Corresponding to the phenomenon in Figure 1b, when the pH value was greater than 5.0, *Oviductus Ranae* protein had very good solubility (the solubility was greater than 87.84 ± 4.16%), while when the pH value was less than 5.0, the solubility of *Oviductus Ranae* protein decreased rapidly. In the pH range of 2.0–3.6, because the pH of the solution was close to the isoelectric point of *Oviductus Ranae* protein, the solubility of the protein was very low, and the minimum solubility measured was 15.95 ± 3.22%.

According to the solubility curve of *Oviductus Ranae* protein, the state of the solution during enzymatic hydrolysis was determined (Figure S1). Among the six different proteases used in this work, the optimal pH value for pepsin was the smallest (pH = 2.0), and the *Oviductus Ranae* protein solution was precipitated in enzymatic hydrolysis in a turbid state. Along with the enzymatic hydrolysis process, the properties of the *Oviductus Ranae* protein solution were also changing. Among them, the most significant change was the enzymatic hydrolysis of pepsin, and the solution gradually became clear and transparent from the turbid state (Figure S1a). Pepsin breaks the specific peptide bond in *Oviductus Ranae* protein, resulting in a relatively small molecular weight of polypeptide, so it is more soluble. On the other hand, the internal groups of *Oviductus Ranae* protein were exposed, which resulted in a change in its isoelectric point. The changed isoelectric point differed greatly from the pH of the solution, and the solubility of the protein increased significantly. The optimal pH values of the other five proteases were all greater than or equal to 6.5. In this environment, the solubility of *Oviductus Ranae* protein was very good, therefore, the solution was always in a clear state during the enzymatic hydrolysis (Figure S1).

### 2.2. DH of Oviductus Ranae Protein

**Figure 2:**
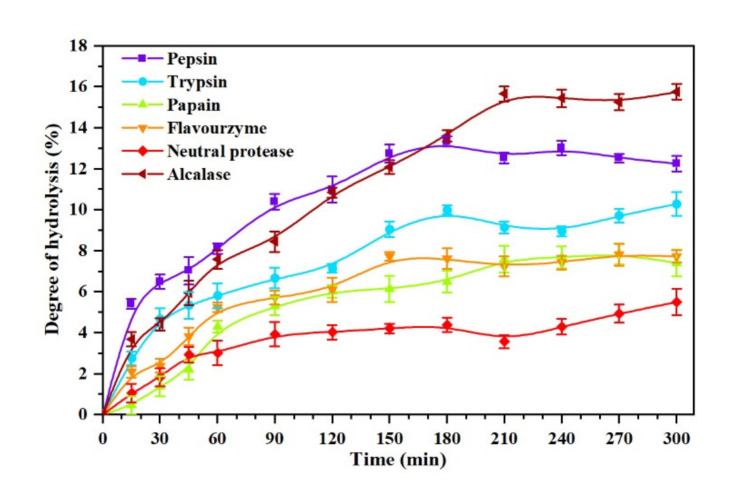
The degree of hydrolysis (DH) curve of the *Oviductus Ranae* protein hydrolyzed by pepsin, trypsin, papain, flavourzyme, neutral protease and alcalase.

DH reflects the changes in peptide bonds during the enzymatic hydrolysis, and it is an important parameter to measure the process of protein enzymolysis [34]. The six kinds of proteases selected in this work were applied to the *Oviductus Ranae* protein, and the change processes of DH at different hydrolysis times are shown in Figure 2. Overall, the DH of *Oviductus Ranae* protein showed a similar trend under the action of different proteases. After a period of significant increase, the DH tended to be stable. However, there were significant differences in the maximum DH and the hydrolysis equilibrium time of the substrate. Specifically, under the action of different proteases, the maximum DH (time to reach hydrolysis equilibrium) of *Oviductus Ranae* protein substrate was 15.65 ± 0.37% (210 min of alcalase hydrolysis), 13.32 ± 0.24% (180 min of pepsin hydrolysis), 9.99 ± 0.24% (180 min of trypsin hydrolysis), 7.71 ± 0.21% (150 min of flavourzyme hydrolysis), 7.58 ± 0.66% (210 min of papain hydrolysis) and 5.48 ± 0.64% (neutral protease hydrolysis 300 min). Among them, the *Oviductus Ranae* protein hydrolyzed by pepsin and alcalase had a relatively high DH, which indicated that these two proteases had more extensive hydrolysis efficacy over the other proteases. This situation was probably related to the characteristics of these two enzymes. For the most suitable working environment of pepsin, the temperature was relatively mild (37 °C), but its pH value was 2.0, which is a strong acidic condition (a relatively extreme reaction condition) for the substrate of the *Oviductus Ranae* protein. In this case, the substrate may have undergone acid hydrolysis, resulting in a relatively high DH. Alcalase is a member of the serine S8 endoproteinase family, which has a broader specificity [35,36]. Compared with other proteases, alcalase had more binding sites on the *Oviductus Ranae* protein, so it showed higher hydrolysis capacity. Since the improvement of the DH is conducive to the release of antioxidant functional groups in proteins, pepsin and alkaline protease have relatively high advantages in the production of antioxidant peptides of *Oviductus Ranae* protein only considering the DH.

### 2.3. 1,1-Diphenyl-2-picrylhydrazyl (DPPH) Radical Scavenging Activity

**Figure 3:**
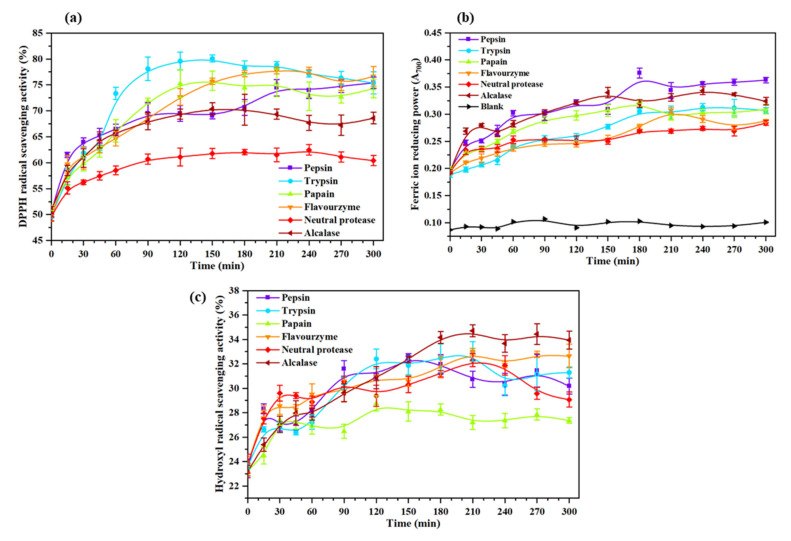
Antioxidant activity of hydrolysates of the *Oviductus Ranae* protein. All data were measured three times in parallel, and all experimental data were expressed in the form of mean ± standard deviation (SD). (**a**) DPPH radical scavenging activity of *Oviductus Ranae* protein and its enzymatic hydrolysates. (**b**) Reducing power of *Oviductus Ranae* protein and its enzymatic hydrolysates. (**c**) Hydroxyl radical scavenging activity of *Oviductus Ranae* protein and its enzymatic hydrolysates.

The curve of DPPH radical scavenging activity of *Oviductus Ranae* protein hydrolysate during enzymatic hydrolysis is shown in Figure 3a. The hydrolysis time (0 min) in Figure 3a corresponded to the *Oviductus Ranae* protein without enzymolysis. It could be observed that *Oviductus Ranae* protein had a certain degree of antioxidant activity before enzymatic hydrolysis, and its DPPH radical scavenging activity was determined to be 50.22 ± 0.69%. Based on the specificity of protease action, the DPPH radical scavenging activity of different hydrolysates was significantly different. After enzymatic hydrolysis of trypsin for 150 min, the DPPH radical scavenging activity of the hydrolysate reached the maximum value of 80.10 ± 0.65%, and compared with the *Oviductus Ranae* protein without enzymolysis, it had the largest improvement. However, after enzymatic hydrolysis of neutral protease for 240 min, the DPPH radical scavenging activity of the hydrolysate reached the maximum value of 62.47 ± 1.02%, and the increase was minimal compared to the *Oviductus Ranae* protein without enzymolysis. The maximum DPPH radical scavenging activities of the other four protease hydrolysates (the enzymatic hydrolysis time corresponding to the maximum value) were 77.75 ± 0.63% (flavourzyme, 210 min), 75.82 ± 1.86% (papain, 150 min), 75.42 ± 1.07% (pepsin, 300 min) and 70.40 ± 1.15% (alcalase, 150 min). Along with the progress of enzymatic hydrolysis, the DPPH radical scavenging activities of various hydrolysates were improved. This is because the complete *Oviductus Ranae* protein is hydrolyzed into various peptide products with smaller molecular weight. Some peptide segments as electron donors can react with DPPH free radicals, converting DPPH into more stable products and terminating the free radical chain reaction, so it shows higher antioxidant activity [37,38].

### 2.4. Reducing Power

The method to determine the reducing power of protein and polypeptide by producing Prussian blue usually takes the value of absorbance as an indicator. The greater the absorbance indicates the stronger reducing power of the sample [39]. The curve of reducing power of the *Oviductus Ranae* protein and its enzymatic hydrolysates is shown in Figure 3b. Compared with the blank group (A_700_ = 0.096 ± 0.006), the absorbance of *Oviductus Ranae* protein without enzymolysis was 0.194 ± 0.003, showing considerable reducing power. Comparing the maximum reducing power of different enzymatic hydrolysates, the product had the strongest reducing power after enzymatic hydrolysis of pepsin for 180 min, and its corresponding absorbance value A_700_ was 0.376 ± 0.009. Under the action of other proteases, the maximum increases in reducing power were in turn alcalase (A_700_ was 0.344 ± 0.007 after 240 min of enzymolysis), papain (A_700_ was 0.323 ± 0.003 after 180 min of enzymolysis), trypsin (A_700_ was 0.313 ± 0.007 after 240 min of enzymolysis), flavourzyme (A_700_ was 0.303 ± 0.005 after 210 min of enzymolysis), and neutral protease (A_700_ was 0.284 ± 0.005 after 120 min of enzymolysis). It could be attributed to the exposure of the electron-dense amino acid side chain groups during the process of protein fracture [40], thus the reducing activity of the enzymatic hydrolysates was improved. In the process of pepsin hydrolysis, the hydrolysate might provide more additional electronic sources, so it showed relatively high reducing activity [40].

### 2.5. Hydroxyl Radical Scavenging Activity

The hydroxyl radical is the most active radical, which can easily react with a variety of biological molecules and seriously harm the health of organisms. To study its antioxidant properties better, Figure 3c shows the hydroxyl radical scavenging activity of *Oviductus Ranae* protein and its enzymatic hydrolysates. Specifically, in the absence of enzymolysis, the hydroxyl radical scavenging activity of *Oviductus Ranae* protein was 23.53 ± 0.24%. After enzymatic hydrolysis with six kinds of proteases, the maximum hydroxyl radical scavenging activities that could be achieved by various enzymatic hydrolysates were in turn 34.72 ± 0.49% (210 min of alcalase hydrolysis), 33.01 ± 0.81% (210 min of trypsin hydrolysis), 32.99 ± 0.27% (210 min of flavourzyme hydrolysis), 32.58 ± 0.27% (150 min of pepsin hydrolysis), 32.30 ± 0.53% (210 min of neutral protease hydrolysis) and 28.76 ± 0.62% (120 min of papain hydrolysis). Similar to the hydrolysate of *hoki (Johnius belengerii)* protein [41] and *Sphyrna lewini* muscle protein [42], due to the exposure of antioxidant groups in protein, enzymolysis effectively enhanced the ability of the *Oviductus Ranae* protein hydrolysate to inhibit hydroxyl radicals and the ability to protect hydroxyl radical-induced damage. Consistent with the measurement results of DPPH radical scavenging activity and reducing power, the hydroxyl radical scavenging activity of the hydrolysates was increased. From the specific data analysis, the maximum hydroxyl radical scavenging activity of the papain hydrolysate was relatively low, while the maximum values of the other five protease hydrolysates had little difference.

### 2.6. Comprehensive Antioxidant Activity Analysis

**Figure 4:**
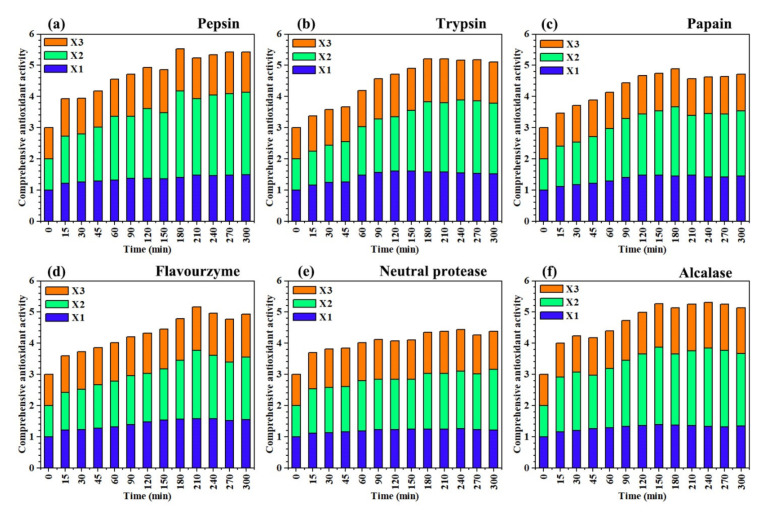
Comprehensive antioxidant activity of enzymatic hydrolysates of different proteases at different times. The experimental data in the figure are the average values of three parallel determinations. (**a**–**f**) The corresponding proteases were pepsin, trypsin, papain, flavourzyme, neutral protease and alcalase. X1: the relative value of DPPH radical scavenging activity, X2: the relative value of reducing power, X3: the relative value of hydroxyl radical scavenging activity.

The comprehensive antioxidant activity (CAA) of different enzymatic hydrolysates was calculated and are presented in Figure 4. Comparing the various parts in Figure 4, pepsin (Figure 4a) had the highest CAA after enzymatic hydrolysis for 180 min (CAA = 5.53). The maximum CAA of alcalase (Figure 4f, 240 min, CAA = 5.30), trypsin (Figure 4b, 180 min, CAA = 5.21) and flavourzyme (Figure 4d, 210 min, CAA = 5.16) were comparable to that of pepsin (CAA > 5.00). However, the CAA of papain (Figure 4c, 150 min, CAA = 4.88) and neutral protease (Figure 4e, 240 min, CAA = 4.43) were relatively weak. The enzymatic hydrolysates of different proteases reached the maximum value of CAA within 180–240 min, indicating that the optimal antioxidant activity of *Oviductus Ranae* protein could not be shown until the enzymatic hydrolysis of *Oviductus Ranae* protein after 3–4 h.

Under the action of different proteases, when the hydrolysis time was less than 3 h, the hydrolysis of the *Oviductus Ranae* protein was not complete enough, the antioxidant functional groups were not completely released, which limited the antioxidant capacity of the hydrolysates. However, when the hydrolysis time was more than 4 h, the DH of protein substrate reached an equilibrium value, the hydrolysates did not change any more, and its antioxidant capacity no longer increased. Therefore, controlling the enzymatic hydrolysis of the protein substrate for 3–4 h was the most ideal hydrolysis time to produce the antioxidant peptides of the *Oviductus Ranae* protein. In various amino acids, histidine, proline, tyrosine and tryptophan are the most important residues in the antioxidant activity of peptides [43]. Pepsin tends to hydrolyze the peptide bonds composed of the aromatic amino acids (including tryptophan and tyrosine) [32], which promote the release of antioxidant functional groups in the *Oviductus Ranae* protein, so its hydrolysate showed the highest CAA among different proteases. At 180 min, the enzymatic hydrolysate of pepsin reached the maximum DH (13.32 ± 0.24%) and the exposed antioxidant active groups no longer increased, so the enzymatic hydrolysate showed the best antioxidant activity. At this time point, the DPPH radical scavenging activity corresponding to the enzymatic hydrolysates was 70.63 ± 1.53%, the absorbance value of reducing power determination was 0.376 ± 0.009, and the hydroxyl radical scavenging activity was 31.96 ± 0.78%.

### 2.7. Correlation Analysis

Through Pearson correlation analysis, Table 1 shows the relationship between hydrolysis time, DH and antioxidant activity of the enzymatic hydrolysates. Pearson correlation coefficient indicates positive correlation when its value is positive. The closer the absolute value is to 1, the greater the correlation. The correlation results showed that there was a significant positive correlation between the hydrolysis time and the DH (*p* < 0.01), indicating that increasing the hydrolysis time was beneficial to improving the DH of *Oviductus Ranae* protein. Hydrolysis time and DH all showed significant positive correlation with the antioxidant activity of the enzymatic hydrolysates. In most cases, the correlation between the DH and the antioxidant activity of the enzymatic hydrolysates was greater than that between the hydrolysis time and the antioxidant activity of the enzymatic hydrolysates, regardless of the types of proteases. These showed that the relationship between the increase of DH and the enhancement of antioxidant activity of enzymatic hydrolysates was closer. The hydrolysate of *Oviductus Ranae* protein had stronger antioxidant activity than intact *Oviductus Ranae* protein, which means that it had a stronger ability to remove or inhibit ROS. Anti-aging is one of the important traditional efficacies of *Oviductus Ranae*, and ROS is the first killer of skin and body to accelerate aging. According to its internal correlation, it can be explained that enzymatic hydrolysis of *Oviductus Ranae* protein is helpful to enhance its traditional anti-aging efficacy, which is the modern theoretical support and optimization of the traditional efficacy of *Oviductus Ranae*.

## 3. Materials and Methods

### 3.1. Chemicals and Samples

**Figure 5:**
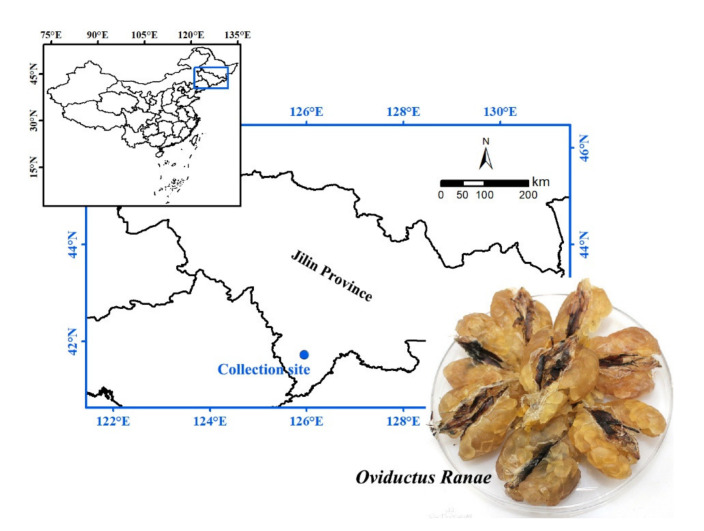
*Oviductus Ranae* sample and its collection site (the main producing areas of Changbai Mountain, Tonghua, Jilin Province, China).

Pepsin (250 U/mg), trypsin (250 U/mg), papain (800 U/mg), flavourzyme (300 U/mg), neutral protease (200 U/mg), alcalase (200 U/mg), serine standard, *o*-phthalaldehyde (OPA), dithiothreitol (DTT) and sodium dodecyl sulfate (SDS) were purchased from Beijing Solarbio Science & Technology Co., Ltd., China. DPPH was purchased from TCI (Shanghai, China) Development Co., Ltd. BCA Protein Assay Kit was purchased from Shanghai Beyotime Biotechnology Co., Ltd., China. Other chemical reagents used in the experiment were analytical grade and purchased from Shanghai Macklin Biochemical Co., Ltd., China. *Oviductus Ranae* samples were collected from the main producing areas of Changbai Mountain, Tonghua, Jilin Province, China (Figure 5), and stored in a refrigerator at −20 °C.

### 3.2. Oviductus Ranae Protein Extraction

The method of extraction of the *Oviductus Ranae* protein was improved on the previous methods [18]. In short, *Oviductus Ranae* was taken out from the refrigerator at −20 °C and dried in a constant temperature drying oven (GZX-9140MBE, Shanghai Boxun Industry & Commerce Co., Ltd., Shanghai, China) at 50 °C for 12 h. The dried *Oviductus Ranae* was crushed into powder by a pulverizer (F2100, Taisite Instrument Co., Ltd., Tianjin, China), and large particles were removed by a 20 mesh (0.850 mm) sieve. Enough *Oviductus Ranae* powder was mixed with ten times volume of *n*-hexane, and the mixture was stirred to degrease for 24 h at room temperature. After filtering, the degreased powder was dried in a constant temperature drying oven at 50 °C for 4 h. Then 8.0 g of the degreased powder was mixed with 800 mL of phosphate buffer solution (PBS, pH 7.4). After one hour of ultrasonic treatment using the ultrasonic cleaner (KQ-300VDE, Kun Shan Ultrasonic Instruments Co., Ltd., Jiangsu, China), it was continuously stirred and extracted for 12 h at room temperature. The mixture was centrifuged at 8000 rpm for 20 min, and the supernatant was collected. The precipitate was repeatedly extracted once the supernatants were combined. The supernatant was put into a dialysis bag (molecular weight cutoff: 8000 Da) and dialyzed in ultrapure water for 24 h. In order to facilitate the determination of DH and antioxidant activity, the protein concentration of the extract was measured by the BCA protein assay kit, and the concentration of *Oviductus Ranae* protein extract was adjusted by PBS to make a final concentration of 1 mg/mL.

### 3.3. Determination of Solubility

In the process of enzymatic hydrolysis, different proteases require different optimal pH values. To determine the solubility of *Oviductus Ranae* protein in different pH environments, the same volume of protein extract was adjusted to different pH values (pH range 1–14) using 2 mol/L HCl or 2 mol/L NaOH solution [29,44]. After stirring at room temperature for 10 min, the solution was centrifuged at 8000 rpm for 20 min, and the supernatant was collected. The BCA protein assay kit was used to measure the protein content in the supernatant, and the protein content in different pH environments was compared with the protein content in the original protein extract of *Oviductus Ranae*. According to Equation (1), the solubility of *Oviductus Ranae* protein in different pH environments was calculated.

(1) $$\mathrm{Protein}\;\mathrm{Solubility}\;(\%)\;=\;\frac{m_{\mathrm{pH}}}{m_{0}}\;\times \;100$$

In the equation, m_pH_ represents the protein content in different pH environments, while m_0_ represents the protein content in the original protein extract without pH adjustment.

### 3.4. Enzymatic Hydrolysis

The pH value and water bath temperature of the *Oviductus Ranae* protein extract were adjusted to the optimal values required for various proteases to perform the enzymatic hydrolysis. The optimal reaction conditions of the proteases recommended by the supplier are shown in Table 2. Then 5% protease (enzyme/protein substrate, *w*/*w*) was added to the solution, and the reaction solution was slowly stirred during the enzymatic hydrolysis reaction [45]. During the enzymatic reaction, the samples were collected every 15 min in the 0–60 min period and then every 30 min in 60–300 min period. The enzymolysis of the protease was terminated by heating the collected samples in the water bath at 95 °C for 10 min. The samples collected at different times were stored in the −20 °C refrigerator for the determination of DH and antioxidant activity.

### 3.5. Determination of DH

The DH of the *Oviductus Ranae* protein can be determined by the OPA method [46,47]. The principle of the OPA method for determining the DH is that OPA can react with free amino groups to form a yellow compound in the presence of DTT, and its characteristic absorption at 340 nm can be detected by a UV–Vis spectrophotometer [47].

Specifically, to prepare 250 mL of OPA solution, 9.525 g of sodium tetraborate decahydrate and 250 mg of SDS were completely dissolved in 180 mL of ultrapure water to make the A1 solution. Then 200 mg of OPA was dissolved in 5 mL of absolute ethanol, and it was transferred to the above A1 solution after being completely dissolved. Afterwards, 220 mg of DTT was added to A1 solution, and the A1 solution was finally made up to 250 mL with ultrapure water. The 0.1 mg/mL of L-serine aqueous solution was used as a standard solution, and the content of SerineNH_2_ in this solution was 0.9516 meqv/L.

After the enzymolysis solution of *Oviductus Ranae* protein (1 mg/mL) was diluted 5 times, 400 μL of the sample dilution solution was added into 3 mL of OPA solution and mixed evenly. The mixture was allowed to stand in the dark for 2 min, and then the absorbance OD_sample_ was recorded at 340 nm using the ultraviolet–visible spectrophotometer (UV754, Shanghai Xinmao Instrument Co., Ltd., Shanghai, China). The L-serine aqueous solution was used instead of the sample as the standard OD_stand_, and ultrapure water was used instead of the sample as the blank OD_blank_. Each data was measured three times in parallel, and all experimental data were expressed in the form of mean ± standard deviation (SD). The DH of the *Oviductus Ranae* protein was calculated according to Equations (2)–(4).

(2) $${\mathrm{SerineNH}}_{2}\;=\;\frac{{\mathrm{OD}}_{\mathrm{sample}}-{\mathrm{OD}}_{\mathrm{blank}}}{{\mathrm{OD}}_{\mathrm{stand}}-{\mathrm{OD}}_{\mathrm{blank}}}\;\times \;\frac{0.9516}{c}$$

(3) $$h\;=\;\frac{{\mathrm{SerineNH}}_{2}-\;\beta }{\alpha }$$

(4) $$\mathrm{DH}\;(\%)\;=\;\frac{h}{h_{\mathrm{tot}}}\;\times \;100$$

In these equations, SerineNH_2_ represents the milliequivalents of amino group per gram of protein; OD_sample_, OD_stand_ and OD_blank_ are the absorbance values of the sample, L-serine standard and blank at 340 nm, respectively; c is the concentration of sample solution (g/L); α and β are 1.00 and 0.40, respectively; h is the number of peptide bonds hydrolyzed; h_tot_ is the total number of peptide bonds of the protein. The value of h_tot_ depends on the amino acid composition of the protein. The h_tot_ value of *Oviductus Ranae* protein is 8.10, based on various amino acid compositions in *Oviductus Ranae* [26,48].

### 3.6. Determination of Antioxidant Activity

Due to the different reaction mechanisms of different free radical systems (such as DPPH, hydroxyl radicals) in the evaluation of antioxidant activity, two or more free radical systems are usually needed to study the antioxidant activity of selected antioxidants in many ways [49,50]. In this work, DPPH radical scavenging activity, reducing power and hydroxyl radical scavenging activity were selected to explore the antioxidant capacity of *Oviductus Ranae* protein and its hydrolysates. Each experiment was measured three times in parallel, and all experimental data were expressed in the form of mean ± SD.

#### 3.6.1. DPPH Radical Scavenging Activity

DPPH is a stable free radical with nitrogen as the center, and it is the most commonly used reagent for the determination of an antioxidant model in vitro [51]. According to the method described in the literature [52], 2 mL of DPPH absolute ethanol solution (0.1 mM DPPH in absolute ethanol) was mixed with 2 mL of enzymolysis solution. After reacting for 20 min at room temperature in the dark, the reaction solution was centrifuged at 5000 rpm for 10 min. The absorbance of the supernatant A_S_ was determined at 517 nm. 2 mL of sample solvent PBS was used instead of the sample as the blank group A_0_. The DPPH radical scavenging activity was calculated according to Equation (5).

(5) $$\mathrm{DPPH}\;\mathrm{radical}\;\mathrm{scavenging}\;\mathrm{activity}\;(\%)\;=\;\frac{A_{0}-{\;A}_{s}}{A_{0}}\;\times \;100$$

#### 3.6.2. Determination of Reducing Power

The antioxidant (reductant) can reduce Fe^3+^ in K_3_[Fe(CN)_6_] to Fe^2+^ through its own reduction power, and Fe^2+^ further reacts with FeCl_3_ to form Prussian blue (Fe_4_[Fe(CN)_6_]_3_) with the maximum absorbance at 700 nm, so the absorbance of the reaction mixture at 700 nm can indirectly reflect the reduction ability of the antioxidant [39]. The specific operation was performed according to the methods described in the literature [52,53]. In brief, 1 mL of enzymolysis solution was mixed with 2 mL of PBS (pH 6.6) and 2 mL of K_3_[Fe(CN)_6_] solution (1%, *w*/*v*), and then the mixture was put into the water bath at 50 °C for 30 min. After being taken out from the water bath, 2 mL of trichloroacetic acid solution (TCA, 10%, *w*/*v*) was added to the mixture. Then the mixture was centrifuged at 3000 rpm for 10 min. Finally, 2 mL of the supernatant was transferred, and mixed with 2 mL of ultrapure water and 0.5 mL of FeCl_3_ solution (0.1%, *w*/*v*). After 10 min reaction at room temperature, the absorbance at 700 nm was measured. The increase of the absorbance of the reaction mixture indicates a stronger reducing power.

#### 3.6.3. Hydroxyl Radical Scavenging Activity

Hydroxyl radical scavenging activity is an important indicator of the antioxidant activity of health products and medicines [49]. In the method, the hydroxyl radicals were produced by the Fenton reaction between H_2_O_2_ and Fe^2+^, and the Fe^2+^ in the aqueous solution of *o*-phenanthroline-Fe^2+^ was oxidized to Fe^3+^, resulting in a decrease in absorbance at 536 nm [54]. The degree of inhibition of the 536 nm absorbance decline rate of the sample reflected the hydroxyl radical scavenging activity of the samples [55]. According to the measuring principle, 1 mL of *o*-phenanthroline absolute ethanol solution (1.5 mM *o*-phenanthroline in absolute ethanol) was mixed with 1 mL of PBS buffer (pH 7.4), and 1 mL of sample solution was added. Mixing thoroughly, 1 mL of FeSO_4_ solution (1.5 mM) was added and the solution was mixed again. Then 1 mL of H_2_O_2_ (0.02%, *v*/*v*) was added to the solution. After reacting for 60 min in a constant temperature water bath at 37 °C, the absorbance value of the sample mixed solution measured at 536 nm was A_S_. Ultrapure water was used instead of H_2_O_2_ as the control group, and its absorbance value A_n_ was measured. Ultrapure water was used instead of the sample as the blank group, and its absorbance value A_0_ was measured. The hydroxyl radical scavenging rate of the antioxidant was calculated according to Equation (6).

(6) $$\mathrm{Hydroxyl}\;\mathrm{radical}\;\mathrm{scavenging}\;\mathrm{activity}\;(\%)\;=\;\frac{A_{s}-A_{0}}{A_{n}-A_{0}}\;\times \;100$$

#### 3.6.4. Comprehensive Analysis of Antioxidant Activity

Due to the different mechanisms of different antioxidant experiments, it is difficult to make a comprehensive evaluation [49]. To unify the comparative criteria of antioxidant activity, the CAA of *Oviductus Ranae* protein and its enzymatic hydrolysates was defined and calculated according to Equation (7).

(7) $$\mathrm{CAA}\;=\;\frac{{X1}_{\min}}{{X1}_{0}}\;+\;\frac{{X2}_{\min}}{{X2}_{0}}\;+\;\frac{{X3}_{\min}}{{X3}_{0}}$$

In this equation, X1_min_, X2_min_ and X3_min_ are the DPPH radical scavenging activity, reducing power, and hydroxyl radical scavenging activity of the enzymatic hydrolysates of specific hydrolysis time; X1_0_, X2_0_ and X3_0_ are DPPH radical scavenging activity, reducing power and hydroxyl radical scavenging activity corresponding to the hydrolysis time of 0 min (i.e., the *Oviductus Ranae* protein without enzymolysis).

### 3.7. Correlation Analysis

Correlation analysis is a statistical method used to evaluate the strength of a relationship between two or more random variables [56]. In this work, Pearson correlation analysis was used to examine the correlation between the hydrolysis time, DH and the antioxidant activity of the hydrolysates. Statistical analysis was performed using SPSS software (version 25.0, SPSS Inc, Chicago, IL, USA). The levels of significant and highly significant difference were *p* < 0.05 and *p* < 0.01, respectively.

## 4. Conclusions

This work evaluated the antioxidant activity of the hydrolysates of *Oviductus Ranae* protein extracts under different proteases and DH, which provide references for the selection of the most suitable proteases and its corresponding DH for the production of *Oviductus Ranae* protein antioxidant peptides. In the analysis of antioxidant activity, the enzymatic hydrolysates of *Oviductus Ranae* protein showed better antioxidant activity than the protein without enzymolysis, and the optimal hydrolysis time of different proteases was obtained between 3–4 h. Among them, the protein hydrolysate which was hydrolyzed by pepsin for 180 min had the strongest comprehensive antioxidant activity, so pepsin was considered to be the most suitable protease among the six proteases for enzymatic hydrolysis of *Oviductus Ranae* protein.

In conclusion, the typical edible proteases were used to hydrolyze *Oviductus Ranae* protein in this work, which not only ensured the edible safety of the hydrolysates, but also filled in the gap of protease selection in the hydrolysis of *Oviductus Ranae* protein, and finally provided a theoretical basis for the further research and development of *Oviductus Ranae* protein hydrolytic oral liquid, protein hydrolysate powder and other related antioxidant health foods. At the same time, the traditional anti-aging efficacy of *Oviductus Ranae* was closely related to the removal of ROS, and its hydrolysates had better antioxidant activity, which also provided indirect support for improving its traditional efficacy.

## Figures and Tables

**Table 1:** Correlation between hydrolysis time, DH of *Oviductus Ranae* protein and antioxidant activity of enzymatic hydrolysates.

Protease		Time	DH	DPPH	Reducing Power	OH	CAA
Pepsin	Time	1.000	0.831 **	0.864 **	0.900 **	0.679 *	0.889 **
	DH	0.831 **	1.000	0.952 **	0.945 **	0.934 **	0.973 **
Trypsin	Time	1.000	0.892 **	0.717 **	0.954 **	0.772 **	0.903 **
	DH	0.892 **	1.000	0.902 **	0.945 **	0.902 **	0.965 **
Papain	Time	1.000	0.916 **	0.795 **	0.829 **	0.563 *	0.809 **
	DH	0.916 **	1.000	0.947 **	0.945 **	0.714 **	0.942 **
Flavourzyme	Time	1.000	0.873 **	0.875 **	0.918 **	0.876 **	0.917 **
	DH	0.873 **	1.000	0.976 **	0.902 **	0.936 **	0.946 **
Neutral protease	Time	1.000	0.864 **	0.745 **	0.867 **	0.572 *	0.815 **
	DH	0.864 **	1.000	0.914 **	0.911 **	0.711 **	0.905 **
Alcalase	Time	1.000	0.952 **	0.689 **	0.808 **	0.929 **	0.839 **
	DH	0.952 **	1.000	0.854 **	0.919 **	0.988 **	0.949 **

DH: degree of hydrolysis; DPPH: DPPH radical scavenging activity; ·OH: hydroxyl radical scavenging activity; CAA: comprehensive antioxidant activity; * *p* < 0.05; ** *p* < 0.01.

**Table 2:** The origin of various proteases and their enzymatic conditions.

Protease	Origin	Optimal pH	Optimal Temperature (°C)
Pepsin	Porcine gastric mucosa	2.0	37
Trypsin	Porcine pancreas	8.0	37
Papain	Papaya plant	6.5	55
Flavourzyme	*Aspergillus oryzae*	6.5	50
Neutral protease	*Bacillus subtilis*	7.0	55
Alcalase	*Bacillus licheniformis*	10.0	45

## Data Availability

The data presented in this study are available in the article and supplementary materials.

## Supplementary Materials

The following are available online, Figure S1: The state of *Oviductus Ranae* protein solution in different time periods (0–300 min) during enzymatic hydrolysis. The six kinds of proteases correspond to (a) pepsin, (b) trypsin (c) papain (d) flavourzyme (e) neutral protease, and (f) alcalase.

## Sample Availability

Samples of the compounds are not available from the authors.
