# Supplementary material for: Antioxidant Activity Evaluation of Oviductus Ranae Protein Hydrolyzed by Different Proteases
Source: Molecules. 2021 Mar 15;26(6):1625. doi: 10.3390/molecules26061625 (PMC8002033; doi:10.3390/molecules26061625)
Supplement: Supplementary file 1 [file molecules-26-01625-s001.pdf]

Supplementary Materials

Antioxidant Activity Evaluation of *Oviductus Ranae* Protein Hydrolyzed by Different Proteases

Shihan Wang [1,*], Yuanshuai Gan [2], Xinxin Mao [1], Hong Kan [1], Nan Li [2], Changli Zhang [2], Zhihan Wang [3] and Yongsheng Wang [2,*]

[1] College of Chinese Medicinal Materials, Jilin Agricultural University, Changchun, Jilin 130118, China; shihanw@jlau.edu.cn (S.W.); maomaoheniunai@126.com (X.M.); kanhong@vip.163.com (H.K.)
[2] School of Pharmaceutical Sciences, Jilin University, Changchun, Jilin 130021, China; ganys18@mails.jlu.edu.cn (Y.G.); lin20@mails.jlu.edu.cn (N.L.); clzhang20@mails.jlu.edu.cn (C.Z.); wys@jlu.edu.cn (Y.W.)
[3] Department of Physical Sciences, Eastern New Mexico University, Portales, NM 88130, USA; zhihan.wang@enmu.edu (Z.W.)
* Correspondence: shihanw@jlau.edu.cn (S.W.); wys@jlu.edu.cn (Y.W.)

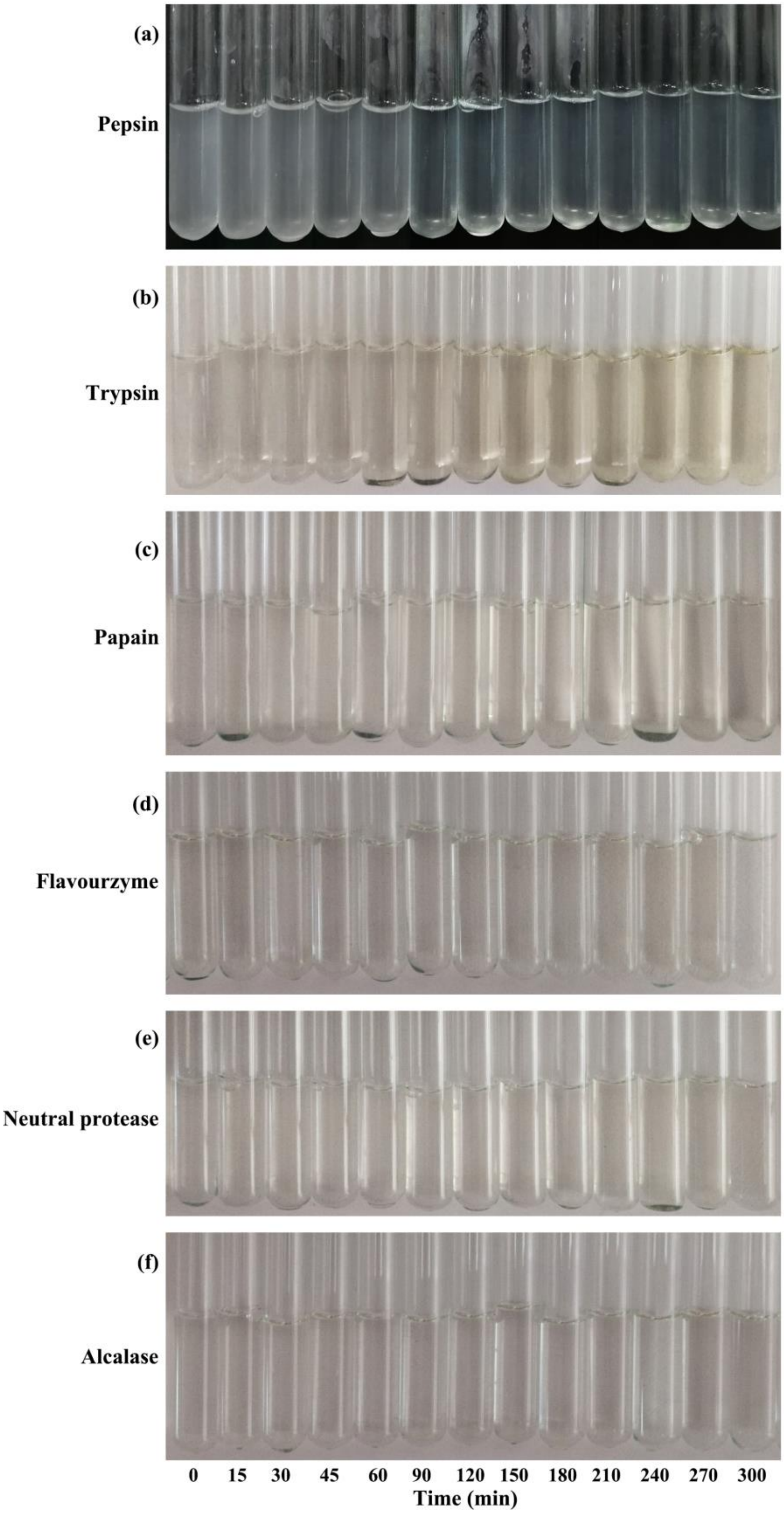

Figure S1. The state of *Oviductus Ranae* protein solution in different time periods (0 – 300 min) during enzymatic hydrolysis. The six kinds of proteases correspond to (a) Pepsin, (b) Trypsin (c) Papain (d) Flavourzyme (e) Neutral protease, and (f) Alcalase.
